# Draft Genome Sequences of Sporulating (CIDEFI-213) and Nonsporulating (CIDEFI-212) Strains of Stemphylium lycopersici

**DOI:** 10.1128/MRA.00960-18

**Published:** 2018-08-16

**Authors:** Rocio Medina, Mario Emilio Ernesto Franco, Gustavo Lucentini, Mario Carlos Nazareno Saparrat, Pedro Alberto Balatti

**Affiliations:** a Centro de Investigaciones de Fitopatología (CIDEFI), Facultad de Ciencias Agrarias y Forestales, Universidad Nacional de La Plata, La Plata, Buenos Aires, Argentina; b Instituto de Fisiología Vegetal (INFIVE-CCT La Plata-CONICET), Facultad de Ciencias Agrarias y Forestales, Facultad de Ciencias Naturales y Museo, Universidad Nacional de La Plata, La Plata, Buenos Aires, Argentina; c Instituto Carlos Spegazzini, Facultad de Ciencias Naturales y Museo, Universidad Nacional de La Plata, La Plata, Buenos Aires, Argentina; d Cátedra de Microbiología Agrícola, Facultad de Ciencias Agrarias y Forestales, Universidad Nacional de La Plata, La Plata, Buenos Aires, Argentina; Georgia Institute of Technology

## Abstract

Stemphylium lycopersici (Pleosporales) is a pathogenic fungus found on a broad range of plant hosts. It is one of the causal agents of gray leaf spot disease in tomato that causes severe yield reductions and economic losses worldwide. Here, we present the draft genome sequences and the gene annotations of two strains of S. lycopersici, nonsporulating strain CIDEFI-212 and sporulating strain CIDEFI-213.

## Abstract (precis)

Stemphylium lycopersici (Pleosporales) is a pathogenic fungus found on a broad range of plant hosts. It is one of the causal agents of gray leaf spot disease in tomato that causes severe yield reductions and economic losses worldwide.

## ANNOUNCEMENT

The genus Stemphylium includes pathogenic, saprotrophic, and endophytic species that are widely distributed in nature (1–4). Stemphylium lycopersici is one of the causative agents of gray leaf spot disease in tomato (5). In a previous study, we reported the draft genome sequence of S. lycopersici strain CIDEFI-216, a virulent and sporulating isolate (6, 7). The purpose of this work was to obtain the draft genome sequences of two strains that proved to be less virulent than isolate CIDEFI-216 (6). While strain CIDEFI-213 sporulates under the conditions assayed on potato dextrose agar (PDA) (7) and V8 medium, strain CIDEFI-212 does not.

Strains CIDEFI-212 and CIDEFI-213 were isolated from tomato cultivar Elpida that presented typical symptoms of gray leaf spot disease (7). Fungal cultures were kept on PDA medium supplemented with kanamycin (40 μg/ml). Total genomic DNA was isolated from 7-day-old monosporic cultures following the protocol of the Wizard genomic DNA purification kit (Promega), with a slight modification involving two extractions, one with phenol-chloroform-isoamyl alcohol (25:24:1 [vol/vol/vol] [pH 7.8 to 8]) and the other one with chloroform-isoamyl alcohol (24:1 [vol/vol]), before alcohol precipitation (8). The quality of the DNA was assessed as described by Franco et al. (6). Libraries were prepared with the TruSeq Nano DNA library preparation kit. Sequencing of 2 × 100-bp paired-end reads was performed using an Illumina HiSeq 4000 sequencing system at Macrogen Co. (Seoul, South Korea). Reads were error corrected and *de novo* assembled using SPAdes software version 3.11.1 (9). Scaffolds of length equal to or greater than 1,000 bp were selected. Structural and functional annotations were made as previously described (7, 10–13).

There were 39,920,136 reads for sample CIDEFI-212 and 32,506,774 reads for CIDEFI-213. The assembled sequences resulted in 598 and 787 contigs, respectively. The draft genome sequence of CIDEFI-212 (*N*_50_, 280,402 bp) had a total consensus length of 34,164,311 bp (GC content, 51.41%), with an average coverage of 33.3×, whereas the draft genome sequence of CIDEFI-213 (*N*_50_, 185,479 bp) had a total consensus length of 34,995,662 bp (GC content, 51.20%), with an average coverage of 41.7×.

Within the CIDEFI-212 genome, 9,024 protein-coding genes were predicted; among them, 5,586 were assigned GO terms, 1,151 with EC numbers, and 7,100 with InterPro codes. Additionally, 115 tRNAs and 33 rRNAs were found. In the genome of CIDEFI-213, 9,079 protein-coding genes were predicted; 6,787 were assigned GO terms, 1,560 with EC numbers, and 7,131 with InterPro codes. Finally, this genome contains 103 tRNAs and 34 rRNAs.

The draft genome sequences presented in this study, as well as that of CIDEFI-216, will be helpful tools for comparing the genomes of S. lycopersici strains that differ in virulence and in their ability to sporulate under a wide array of conditions. This will help us to understand the molecular basis of pathogenesis, as well as of virulence and sporulation.

### Data availability.

The assembled draft genome sequences have been deposited at DDBJ/ENA/GenBank under the accession numbers QGDG00000000 (CIDEFI-212) and QGDH00000000 (CIDEFI-213). The versions described in this paper are versions QGDG01000000 (CIDEFI-212) and QGDH01000000 (CIDEFI-213).
